# Evaluation of a Novel Finite Element Model of Active Contraction in the Heart

**DOI:** 10.3389/fphys.2018.00425

**Published:** 2018-04-23

**Authors:** Xiaoyan Zhang, Zhan-Qiu Liu, Kenneth S. Campbell, Jonathan F. Wenk

**Affiliations:** ^1^Department of Mechanical Engineering, University of Kentucky, Lexington, KY, United States; ^2^Department of Physiology, University of Kentucky, Lexington, KY, United States; ^3^Department of Surgery, University of Kentucky, Lexington, KY, United States

**Keywords:** cross-bridge kinetics, velocity-dependence, relaxation, sarcomere lengthening, left ventricle

## Abstract

Finite element (FE) modeling is becoming a widely used approach for the investigation of global heart function. In the present study, a novel model of cellular-level systolic contraction, which includes both length- and velocity-dependence, was implemented into a 3D non-linear FE code. To validate this new FE implementation, an optimization procedure was used to determine the contractile parameters, associated with sarcomeric function, by comparing FE-predicted pressure and strain to experimental measures collected with magnetic resonance imaging and catheterization in the ventricles of five healthy rats. The pressure-volume relationship generated by the FE models matched well with the experimental data. Additionally, the regional distribution of end-systolic strains and circumferential-longitudinal shear angle exhibited good agreement with experimental results overall, with the main deviation occurring in the septal region. Moreover, the FE model predicted a heterogeneous distribution of sarcomere re-lengthening after ventricular ejection, which is consistent with previous *in vivo* studies. In conclusion, the new FE active contraction model was able to predict the global performance and regional mechanical behaviors of the LV during the entire cardiac cycle. By including more accurate cellular-level mechanisms, this model could provide a better representation of the LV and enhance cardiac research related to both systolic and diastolic dysfunction.

## Introduction

Cardiac muscle constitutes the histological foundation of the heart. The contraction of cardiac muscle cells (i.e., myocytes) generates force and propels blood out of the heart chambers into the circulatory system. In general, the active contraction of myocytes involves complex mechanisms. Briefly, upon activation, the influx of Ca^2+^ ions promotes their binding to the troponin C molecules, altering the shape of the troponin complex and exposing the binding sites on the actin monomers to myosin heads. Once attached to the binding sites, the myosin heads undergo conformational changes and generate force, leading to shortening of the sarcomeres, the contractile units of myocytes (Gordon et al., 2000; Kobayashi and Solaro, 2005). *In vitro* cellular experiments have shown that the force generated during myocyte contraction depends on the intracellular concentration of free Ca^2+^ and the length, as well as the shortening velocity, of sarcomeres (ter Keurs et al., 1980; Daniels et al., 1984; Keurs et al., 1988).

Computational modeling is an important approach for studying the function of the heart, especially the left ventricle (LV). Previously, several finite element (FE) models have been established to investigate the muscle contraction of the LV during cardiac systole (Guccione and McCulloch, 1993; Guccione et al., 1993; Hunter et al., 1998). Among those widely used FE models is the simple but computationally efficient time-varying “elastance” model, in which the active fiber stress is computed from a function of peak intracellular Ca^2+^ concentration, time and sarcomere length (Guccione et al., 1993; Kerckhoffs et al., 2007). This model has been used to successfully assess the effects of apical torsion on heart function (Trumble et al., 2011), as well as predict decreased contractility in the border zone myocardium of ovine and human hearts with myocardial infarction (Wenk et al., 2011, 2012). However, this modeling approach does not include the force-velocity relation of cardiac muscle contraction, and neglects to represent the processes occurring at the cellular level.

In addition to the global-level FE models, there are several cellular-level models of myocyte contraction (Trayanova and Rice, 2011). These models were developed based on the Huxley 2-state cross-bridge model (Huxley, 1957), and have been shown to reproduce the typical mechanical features of myocyte contraction under certain experimental conditions (Schneider et al., 2006; Campbell et al., 2008; Rice et al., 2008). Recently, a more flexible myocyte contraction model (called MyoSim) has been developed by Campbell (2014). MyoSim extends the Huxley model by incorporating Ca^2+^ activation, cooperative effects and the effects of interfilamentary movement, which implicitly depend on the length and velocity of sarcomeres during contraction (Campbell, 2014). Moreover, the use of cross-bridge distribution techniques allows MyoSim to predict the mechanical behavior of myocytes under a wider range of experimental conditions as compared to the deformation-based methods (Razumova et al., 1999; Rice et al., 2008). However, cellular-level models are unable to predict the global function of the heart. In this regard, a deformation-based myocyte contraction model (Rice et al., 2008) has been previously adapted into a FE model of the LV to investigate the mechanical properties of a mouse heart (Land et al., 2012). This suggests that the incorporation of an enhanced cellular-level contraction model, i.e., MyoSim, may further improve the behavior of a global-level FE model of ventricular function.

Along this line, the present work introduces a 3D FE implementation of cardiac muscle contraction that was developed based on MyoSim. This allows for the coupling of cellular-level mechanisms into a ventricle-level model. The new method was then validated using animal-specific FE models of the whole LV in rats. Specifically, the results of the models were fit to experimental measures of myocardial strain and ventricular hemodynamics, in order to determine the sarcomeric parameters that govern contraction.

## Materials and methods

### Experimental measurements

In order to assess regional wall deformation in the LVs of healthy rats, 3D cine displacement encoding with stimulated echoes (DENSE) cardiovascular magnetic resonance (CMR) imaging was performed on 5 female Sprague-Dawley rats (~6 months; Harlan, Indianapolis, IN, USA) using a 7T Bruker ClinScan system (Bruker, Ettlingen, Germany; Zhong et al., 2011; Haggerty et al., 2013; Zhang et al., 2017), followed by LV pressure measurements using a pressure transducer (SPR-903, Millar Instruments, Houston, TX, USA; Pacher et al., 2008). End systolic (ES) strains, relative to end diastole (ED), were calculated using the software DENSEanalysis (Spottiswoode et al., 2007). ES LV torsion, represented as the circumferential-longitudinal (CL) shear angle α_*CL*_, was calculated as follows (Rüssel et al., 2009; Zhang et al., 2017):

(1) $$\begin{array}{l} \alpha_{CL}=sin^{-1}\frac{2E_{cl}}{\sqrt{\left(1+2E_{cc})(1+2E_{ll}\right)}} \end{array}$$

In order to generate animal-specific FE models, at a minimally loaded state, the LV myocardium was contoured from the CMR images at early-diastolic filling. These contours where then converted into 3D geometric surfaces. All animal procedures were approved by the Institutional Animal Care and Use Committee at the University of Kentucky and were in agreement with the guidelines by the National Institutes of Health for the care and use of laboratory animals (NIH Publication 85–23, revised 1996).

### Ventricular FE model

**Figure 1:**
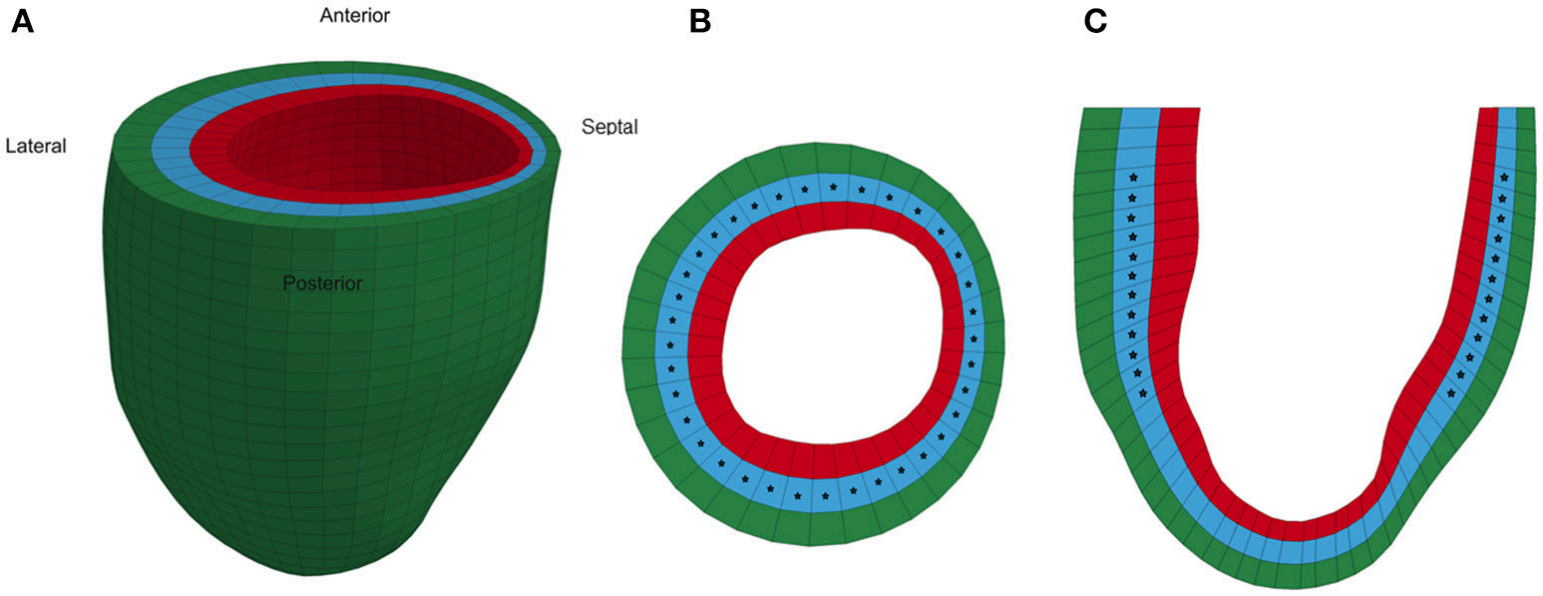
Representative animal-specific FE model of a rat LV. **(A)** Full view of the LV model with the four segments labeled, **(B)** Short axis view of a mid-ventricular slice, and **(C)** Long axis view of a longitudinal slice. Stars represent the points in the model where strain was compared to experimental measurements during the optimization.

**Figure 2:**
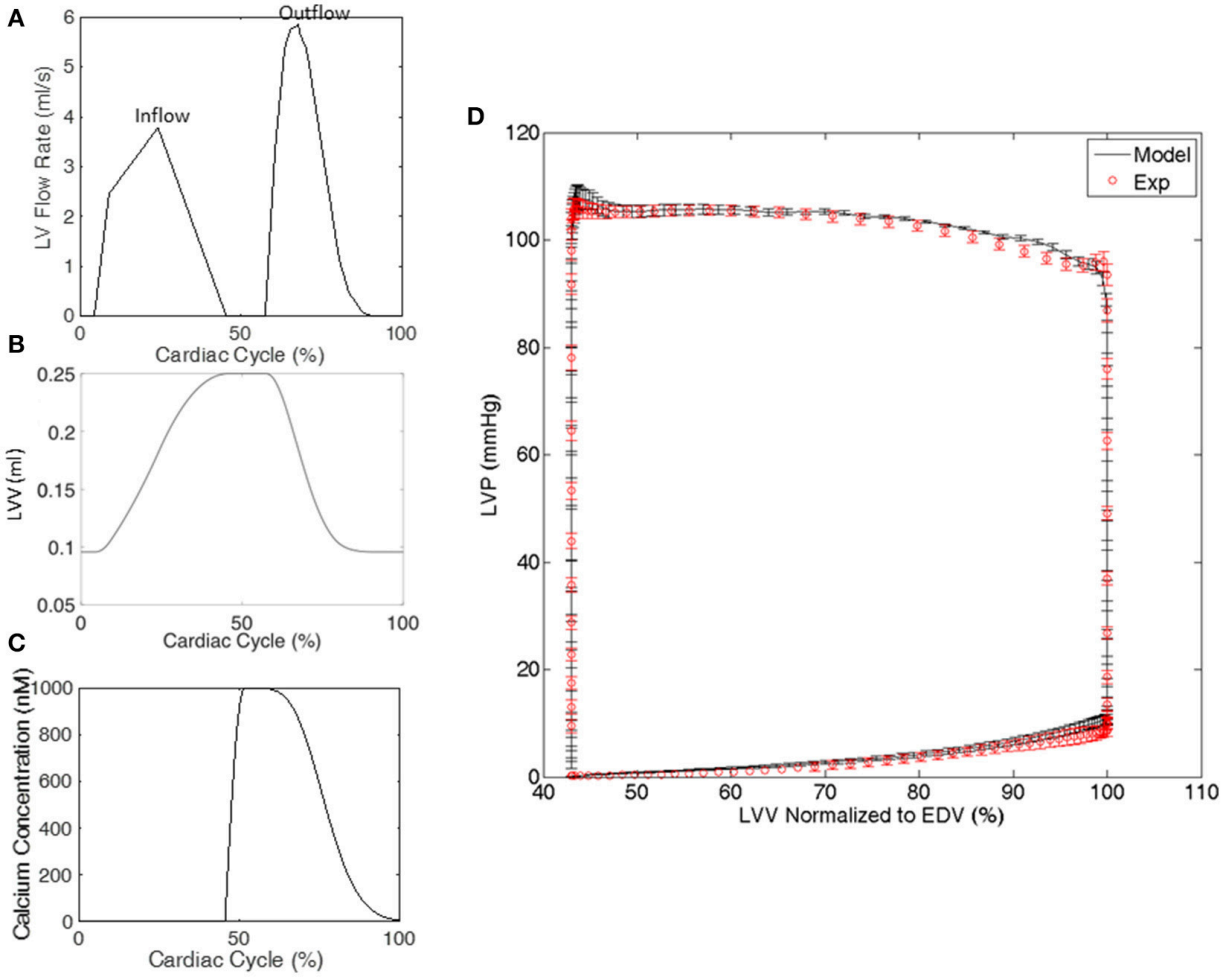
Representative examples from a single rat of **(A)** the volumetric flowrate into and out of the LV used to drive the cardiac cycle in the FE model, **(B)** the LV volume (LVV) generated by the FE model, and **(C)** the calcium transient in the LV model**. (D)** The LV pressure-volume relationship generated by all of the FE models and experiments. LV pressure (LVP) data are mean ± standard error (SE); *n* = 5. LVV data shown in **(D)** were normalized to end diastolic volume (EDV).

Each animal-specific LV FE model was created based on the geometric surface data and size of the corresponding rat LV at early diastole (Figure 1). The FE mesh was produced by filling the myocardial wall with 8-node hexahedral brick elements incorporating a trilinear interpolation scheme (TrueGrid; XYZ Scientific, Inc., Livermore, CA, USA). The myocardium was evenly divided into 3 layers (i.e., epicardium, mid-myocardium, and endocardium; Figure 1; Zhang et al., 2015). The initial sarcomere lengths in each of the three layers, defined in the unloaded reference state of the model, were epi: 1,910 nm, mid: 1,850 nm, and endo: 1,780 nm (Guccione et al., 1993). The helical fiber angles in the epicardium, mid-myocardium, and endocardium were assigned as −60, 0, and 60°, respectively, relative to the circumferential direction. The boundary conditions of the LV were assigned to allow the base to expand/contract radially within the plane, with movement in the direction normal to the plane fully constrained. To simulate the passive filling and ejection of the LV, the volumetric flowrate into and out of the LV was estimated from CMR images (Figures 2A,B) and used to drive the entire cardiac cycle.

The myocardium of the LV was assumed to be nearly incompressible, transversely isotropic, and hyperelastic. The passive stresses were derived from the following strain energy function:

(2) $$\begin{array}{l} W=\frac{C}{2}\{exp[b_{f}E_{11}^{2}+b_{t}\left(E_{22}^{2}+E_{33}^{2}+E_{23}^{2}+E_{32}^{2}\right)\\ \;+b_{fs}(E_{12}^{2}+E_{21}^{2}+E_{13}^{2}+E_{31}^{2})]-1\} \end{array}$$

where *E*_11_ is fiber strain, *E*_22_ is cross-fiber strain, *E*_33_ is radial strain, and the remaining terms are shear strains (Guccione et al., 1993; Wenk et al., 2011). Values for the material constants *b*_*f*_, *b*_*t*_, and *b*_*fs*_ were chosen as 18.48, 3.58, and 1.627, respectively, based on previous studies (Guccione et al., 2001; Zhang et al., 2015). The material constant *C* was adjusted until the LV ED pressure matched the experimentally measured value for each rat. The value was found to be *C* = 0.262 ± 0.082 kPa (mean ± standard error).

**Figure 3:**
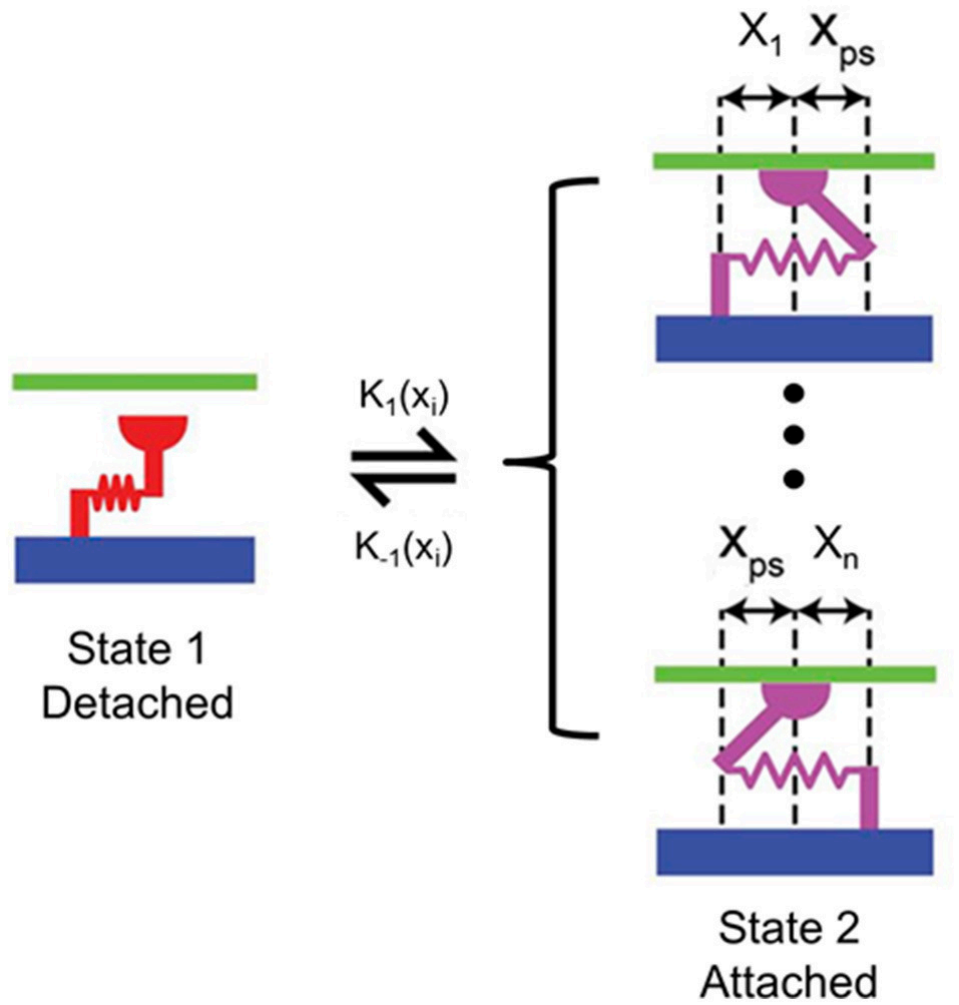
Schematic of the 2-state contraction model based on MyoSim. *x*_1_ to *x*_*n*_ are different lengths of cross-bridges (*n* = 21 in the proposed FE model); *x*_*ps*_ is the cross-bridge length when the myosin head is bound to a directly opposed binding site; *k*_1_ (*x*_*i*_) and *k*_−1_ (*x*_*i*_) are the strain-dependent rate constants for the attachment and detachment transitions, respectively.

The active material properties were derived from the 2-state MyoSim model of striated muscle (Campbell, 2014). Briefly, the myosin heads can switch between detached and attached states, and can form cross-bridges with different lengths ranging from −10 to 10 nm (Figure 3). Particularly, when a myosin head is bound to a directly opposed binding site, the length of the cross-bridge was assigned a value of *x*_*ps*_ (Figure 3). In the current model, the cross-bridges were classified, based on their lengths, into 21 bins with bin-width of 1 nm (i.e., *x*_1_ to *x*_*n*_, *n* = 21; Figure 3). 21 bins were used to maintain an appropriate balance between accuracy and computational efficiency. The population distribution of cross-bridges in the *i*th bin, i.e., the number of myosin heads attached to the thin filament with a cross-bridge length of *x*_*i*_ at time *t*, which is defined as *A*(*x*_*i*_, *t*), was calculated with the following set of 21 coupled ordinary differential equations (ODE) using an explicit 4th-order Runge-Kutta method with adaptive time-step size:

(3) $$\begin{array}{l} \frac{\partial A\left(x_{i},t\right)}{\partial t}=k_{1}\left(x_{i}\right)D\left(t\right)-k_{-1}\left(x_{i}\right)A\left(x_{i},\;t\right)\;\left(i=1,2,3,\ldots ,21\right) \end{array}$$

where *k*_1_ (*x*_*i*_) and *k*_−1_ (*x*_*i*_) are the strain-dependent rate constants for the attachment and detachment transitions, respectively. *D*(*t*) is the number of myosin heads in the detached state and is determined by the following equation:

(4) $$\begin{array}{l} D\left(t\right)=N\left(t\right)-N_{bound} \end{array}$$

where *N*(*t*) is the total number of sites that are activated and available for myosin heads to attach to, and *N*_*bound*_ is the number of sites that have already been occupied by myosin heads and is equivalent to the total number of myosin heads in the attached state (i.e., $\overset{21}{\underset{i=1}{\sum }}A\left(x_{i},t\right)$). To compute the total number of activated sites, *N*(*t*), a forward Euler method was used to solve the following equation:

(5) $$\begin{array}{l} \frac{dN\left(t\right)}{dt}=a_{on}\left[Ca^{2+}\right]\left(N_{overlap}-N\left(t\right)\right)-a_{off}\left({N\left(t\right)-N}_{bound}\right)\\ +k_{plus}N_{bound}-k_{minus}\left(N_{overlap}-N\left(t\right)\right) \end{array}$$

where *a*_*on*_ and *a*_*off*_ are rate constants, *k*_*plus*_ and *k*_*minus*_ are constants defining cooperative effects, and *N*_*overlap*_ is the maximum number of binding sites that heads could potentially interact with and was defined by the relative positions of the thick and thin filaments within each half of a sarcomere, as previously reported (Campbell, 2009). A calcium transient [*Ca*^2+^], which was based on experimentally recorded transients from intact rabbit hearts (Laurita and Singal, 2001), was temporally scaled according to each animal-specific heart rate and used in all 5 FE models in the present study (Figure 2C). The beginning of the transient was synchronized to the beginning of the isovolumic phase in each FE model. To include the effects of interfilamentary movement, linear interpolation was used to displace the population distributions *A*(*x*_*i*_, *t*) by (1/2)Δ*x*, yielding *A*_*s*_(*x*_*i*_, *t*), where Δ*x* is the half-sarcomere length change between time steps (Huxley et al., 1994; Tajima et al., 1994; Campbell, 2014). Since Δ*x* is highly influenced by the velocity of sarcomere shortening/lengthening, the incorporation of interfilamentary movement accounts for, at least in part, the velocity-dependence of force generation.

Finally, the active stress along the fiber direction (*T*) produced by cross-bridges was calculated as follows:

(6) $$\begin{array}{l} T\left(t\right)=\sum_{i\;=1}^{21}\rho k_{cb}A_{s}\left(x_{i},t\right)\left(x_{i}+x_{ps}\right) \end{array}$$

where ρ is the number of myosin heads in a hypothetical half-sarcomere with a cross-sectional area of 1 m^2^, and *k*_*cb*_ is the stiffness of the cross-bridges. To ensure that the FE model generates realistic active stress throughout the entire cardiac cycle, the following conditions were applied within the model framework: (i) *N*(*t*) ≥ 0 at any time; (ii) *N*(*t*) ≥ *N*_*bound*_ at any time point; (iii) *D*(*t*) ≥ 0 at any time; (iv) active fiber stress *T* = 0 when the sarcomere length was shortened to 1.20 μm. These conditions are necessary in order to maintain a physiological response, i.e., not allowing *N*(*t*) to attain a negative value. A summary of parameters used for the active stress calculation is shown in Table 1, which are related to the MyoSim model (Campbell, 2014). In addition, stress components equivalent to 25% of the fiber stress were added to the two cross-fiber directions.

**Table 1:** Parameters for active stress calculation with 2-state model.

**Parameter**	**Definition**	**Value**	**Unit**
ρ	Number of myosin heads in a hypothetical half-sarcomere with a cross-sectional area of 1 m^2^	6.9 × 10^16^	m^−2^
*k*_*cb*_	Stiffness of the cross-bridges	0.001	N/m
*x*_*ps*_	Cross-bridge length when a myosin head is bound to a directly opposed binding site	5	nm
*a*_*on*_	Rate constant	DO^*^	s^−1^nM^−1^
*a*_*off*_	Rate constant	DO^*^	s^−1^
*k*_*plus*_	Constant defining positive cooperative effects	DO^*^	s^−1^
*k*_*minus*_	Constant defining negative cooperative effects	DO^*^	s^−1^
*k*_1_ (*x*_*i*_)	Attachment rate constant	$C_{k}e^{\frac{-k_{cb}{x_{i}}^{2}}{2BT}}$ (DO^*^)	s^−1^nm^−1^
*k*_−1_ (*x*_*i*_)	Detachment rate constant	$k_{d}+k_{db}{x_{i}}^{4}$ (DO^*^)	s^−1^

*x_i_, cross-bridge length as defined in Figure 3 in nm. B, Boltzmann constant (1.381 × 10^- 23^ JK^- 1^). T, the experimental temperature (288 K)*.

DO^*^, Determined by optimization.

Both the passive and active material laws were implemented as a user defined material subroutine in the explicit non-linear FE solver LS-DYNA (Livermore Software Technology Corporation, Livermore, CA, USA).

### Optimization procedure

In order to determine the active material parameters used for the 2-state contraction model, numerical optimization was performed with the software LS-OPT (Livermore Software Technology Corporation, Livermore, CA) as previously described (Wenk et al., 2012; Wang et al., 2016). In the current study, a hybrid technique was employed, which utilized global and local search algorithms. Specifically, simulated annealing was used as a global optimizer to locate the region of the parameter space with the highest probability of containing the best set of parameters. Once this region was identified, the sequential response surface method, which is a gradient-based method, was employed to find the local minimum by iteratively reducing the size of the parameter space until the optimal set of parameters was achieved. A total of 10 parameters were optimized within the ranges initially set according to the values used for unloaded twitch contraction (Table 2) (Campbell, 2014). The goal of the optimization was to minimize the objective function (Φ), which was taken to be the sum of the squared error between experimentally measured data and FE predicted results, and was defined as follows:

(7) $$\begin{array}{l} \Phi =\sum_{n\;=1}^{N}\sum_{i,j\;=1,2,3}{\left(E_{ij,n}-{\bar{E}}_{ij,n}\right)}^{2}+\sum_{m\;=1}^{6}{\left(\frac{P_{m}-{\bar{P}}_{m}}{{\bar{P}}_{m}}\right)}^{2} \end{array}$$

**Table 2:** Optimization results.

		**Initial range**	**Case 1**	**Case 2**	**Case 3**	**Case 4**	**Case 5**
*a*_*on*_		(0.001, 0.04)	0.019	0.020	0.028	0.024	0.021
*a*_*off*_		(100, 1,000)	211	348	309	460	206
*C*_*k*_	*x*_*i*_ > 0	(0, 2,100)	1,544	1,735	1,885	2,085	2,015
	*x*_*i*_ < 0	(0, 2,100)	264	1,345	1,166	1,113	529
*k*_*d*_	*x*_*i*_ > 0	(0, 400)	293	343	280	190	247
	*x*_*i*_ < 0	(0, 400)	195	74	51	290	267
*k*_*db*_	*x*_*i*_ > 0	(0, 50)	26	49	47	42	36
	*x*_*i*_ < 0	(0, 50)	47	19	28	18	19
	*k*_*plus*_	(0, 100)	54	50	40	72	35
	*k*_*minus*_	(0, 20)	12	14	19	18	13
Φ/element (strain)		N/A	0.077	0.078	0.055	0.080	0.076

*x_i_: cross-bridge length as defined in Figure 1 in nm*.

The first term of the objective function represents the errors induced by ES strains, where n is the strain point within the myocardium, N is the total number of strain points (N ≥ 250 points evenly distributed throughout the mid-layer of the FE model), and *E*_*ij*_ and ${\bar{E}}_{ij}$ are FE-predicted and experimentally measured ES strains, respectively. The second term represents the errors due to LV pressures. In this study, 6 pressure points were compared, including pressure at the end of isovolumetric contraction (IVC), three points during systolic ejection (including the peak pressure), ES pressure, and the end of isovolumetric relaxation (IVR). *P*_*m*_ and ${\bar{P}}_{m}$ are the FE-predicted and experimentally measured LV pressures, respectively.

### Single element FE model

In order to confirm that the optimized parameters, which were determined by fitting organ level data, result in reasonable *in vivo* cellular level function, a single element FE model was employed. Specifically, the model was subjected to boundary conditions and calcium levels that replicate the following cellular experiments (de Tombe and Stienen, 2007):

1. Maximum tension generation (*T*_*max*_) at a fixed sarcomere length of 2,300 nm and maximal calcium concentration.
2. Force-calcium relationship, in terms of Calcium sensitivity (*pCa*_*50*_) and Hill coefficient, at a fixed sarcomere length of 2,300 nm.
3. Maximum and minimum tension redevelopment (*k*_*tr*_) at a sarcomere length of 2,300 nm with 20% length release for 20 ms followed by restretch to 2,300 nm.

## Results

The optimizations for all 5 animal cases displayed good convergence, and the values of the 10 active parameters optimized for each case are shown in Table 2. The mean of squared errors between experimentally measured strains and FE predicted data, i.e., Φ/element (strain), is also shown for each case with values <0.1. Using optimized values for the active parameters, the FE models generated a LV pressure-volume (PV) loop that showed strong agreement with experimental results over the entire cardiac cycle, especially during systole (Figure 2D).

**Figure 4:**
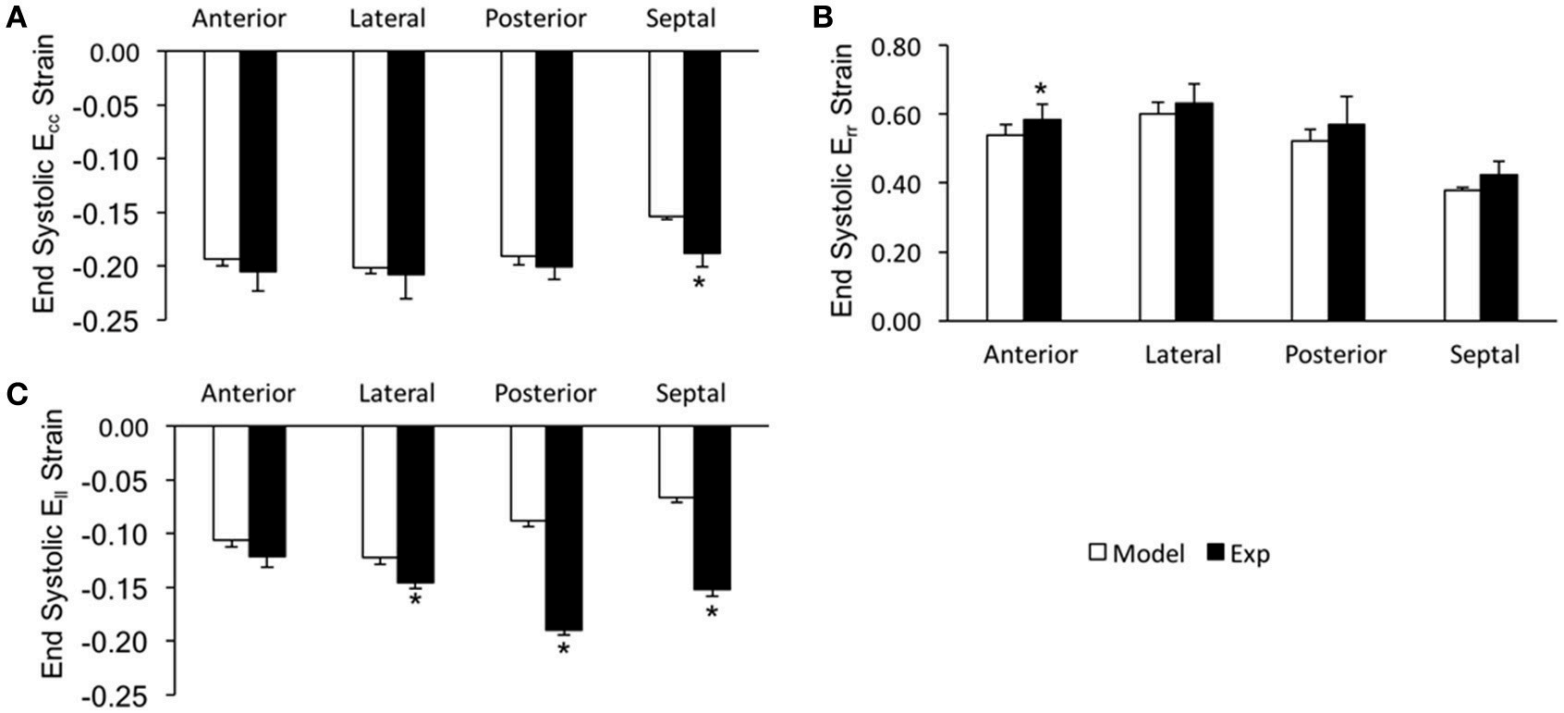
End systolic axial strain components (**A**: E_cc_, **B**: E_rr_, and **C**: E_ll_) averaged at the mid-LV in the mid-myocardial layer. Data are mean ± SE; *n* = 5; ^*^p < 0.05 for comparisons between model-predicted and experiment-derived strains using paired two-tailed *t*-test.

**Figure 5:**
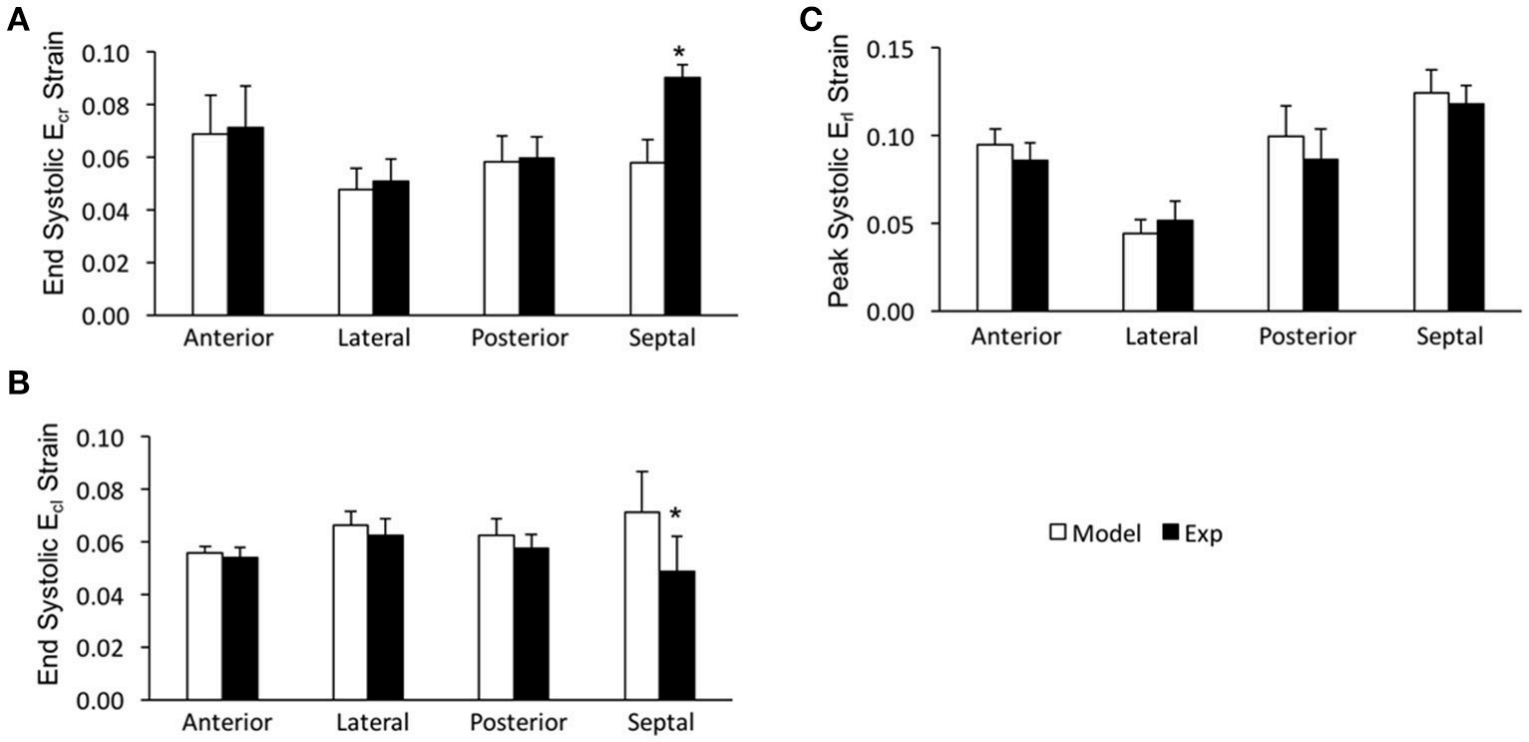
End systolic shear strain components (**A**: E_cr_, **B**: E_cl_, and **C**: E_rl_) averaged at the mid-LV in the mid-myocardial layer. Data are mean ± SE; *n* = 5; ^*^p < 0.05 for comparisons between model-predicted and experiment-derived strains using paired two-tailed *t*-test.

**Figure 6:**
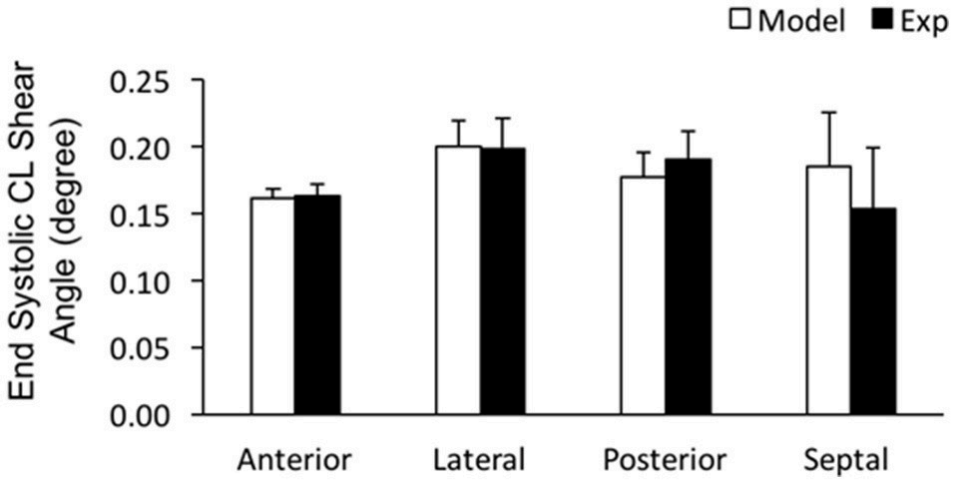
End systolic circumferential-longitudinal (CL) shear angles averaged at the mid-LV in the mid-myocardial layer. Data are mean ± SE; *n* = 5. No significant differences between model-predicted and experiment-derived results using paired two-tailed *t*-test.

In order to assess the accuracy of the FE modeling approach for capturing regional variations in LV wall deformation, all 6 components of ES strain (i.e., E_rr_, E_cc_, E_ll_, E_cl_, E_rl_, and E_cr_) were analyzed within 4 wall segments (i.e., Anterior, Lateral, Posterior, and Septal) in the mid-ventricular region. In terms of the axial components of strain, the FE model predicted similar ES circumferential and radial (E_cc_ and E_rr_) strain distributions in the majority of the mid-myocardium, when compared to the experimental measures, with significant (*p* < 0.05) deviation seen only in the septal and anterior segments, respectively (Figures 4A,B). The FE model, however, significantly (*p* < 0.05) underestimated systolic longitudinal strain (E_ll_) in the lateral, posterior and septal segments of the mid-myocardium (Figure 4C). This could be due to the assumed longitudinal myofiber angle distribution. While there existed some significant (*p* < 0.05) differences between FE predicted values and experimental measures for ES shear strains E_cr_ and E_cl_ (only in the septal segment of the mid-myocardium), the shear strain E_rl_ predicted by the model was comparable with experimental data throughout the entire mid-myocardium (Figure 5). Moreover, the mid-ventricular torsion at ES, represented as CL shear angle, was similar to that estimated from experimental measures over the entire mid-myocardium (Figure 6).

**Figure 7:**
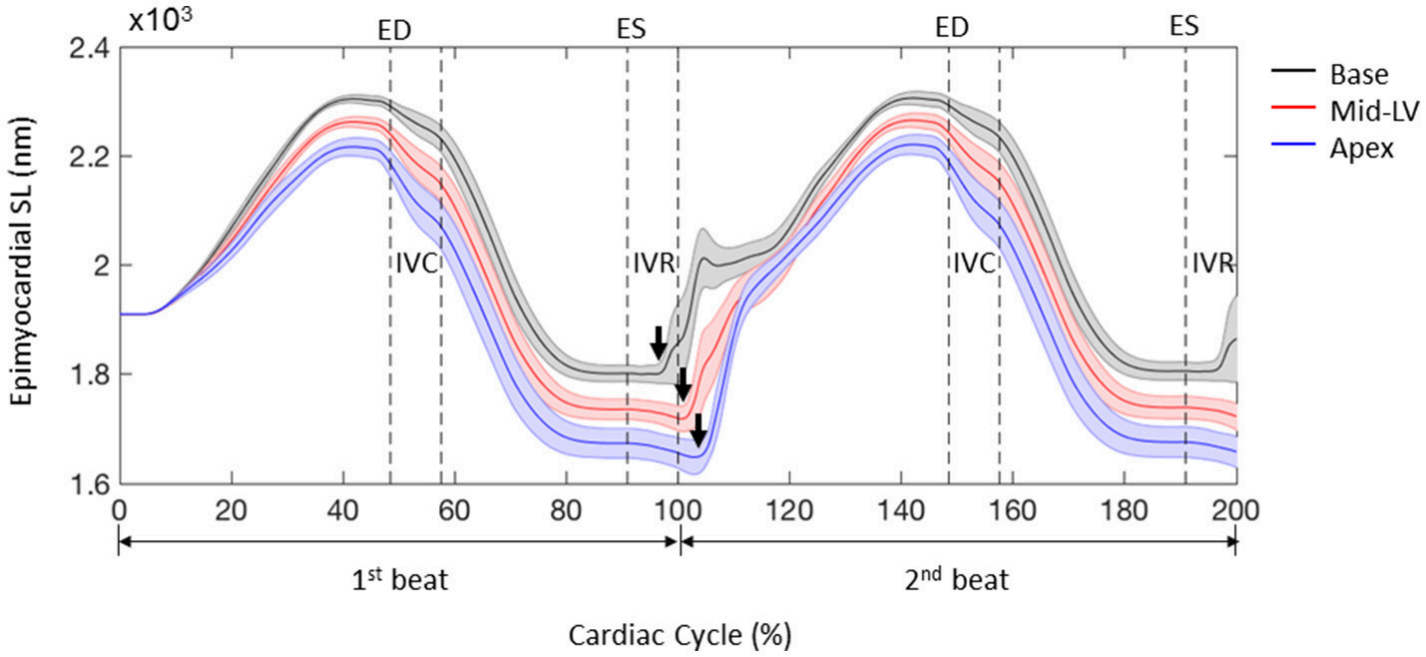
Profiles of sarcomere length (SL) over 2 cardiac cycles obtained from 3 representative elements located at the base, mid-LV, and apex in the lateral epicardium. Data are mean (thick solid lines) ± SE (shaded area); *n* = 5. Note that the simulations start from the unloaded state. Arrows indicate the beginning of re-lengthening.

**Figure 8:**
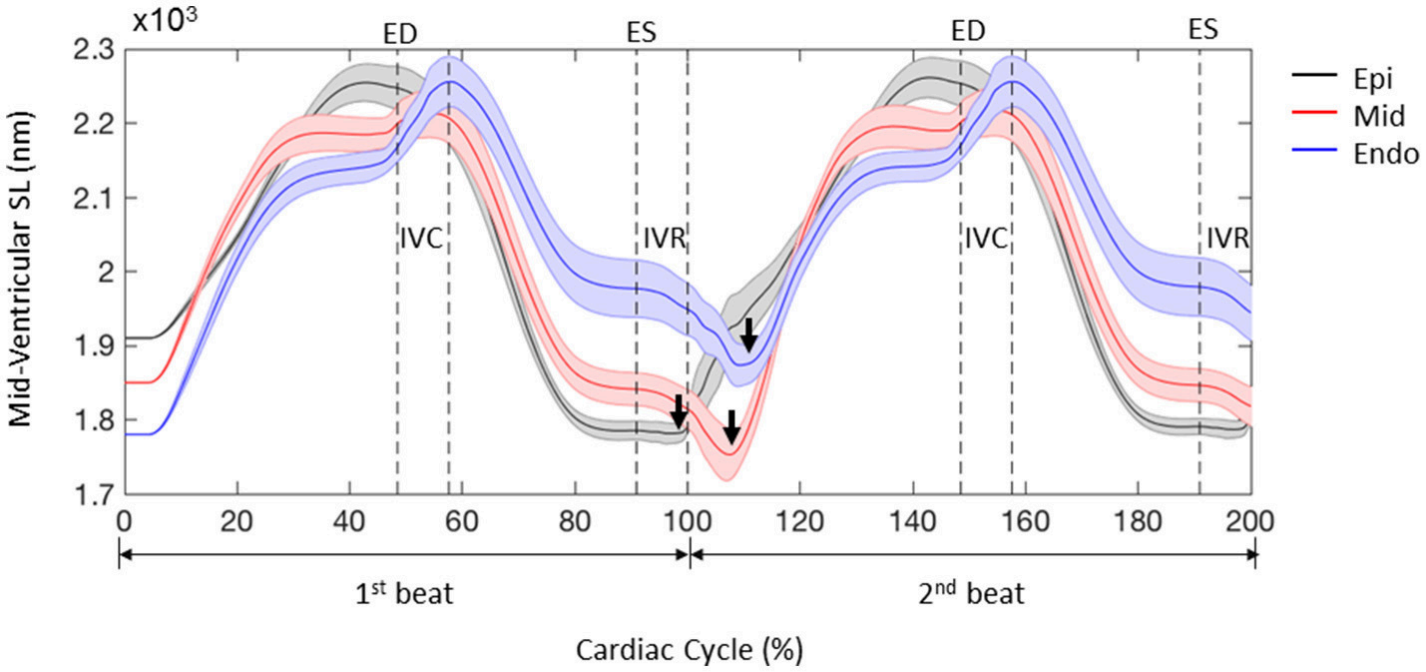
Profiles of sarcomere length (SL) over 2 cardiac cycles obtained from 3 representative elements located in the lateral epicardium (epi), mid-myocardium (mid), and endocardium (endo) at the mid-LV. Data are mean (thick solid lines) ± SE (shaded area); *n* = 5. Note that the simulations start from the unloaded state. Arrows indicate the beginning of re-lengthening.

The FE model predicted sequential relaxation throughout the entire LV myocardium. Specifically, in contrast to the prompt sarcomere re-lengthening that occurred at the base, both the mid-ventricle and apex exhibited prolonged post-systolic shortening (PSS) and delayed re-lengthening and relaxation in the epicardial layer (Figure 7, arrows indicate the beginning of re-lengthening). This sequential re-lengthening and relaxation was mitigated in the mid-myocardium, and eventually reversed in the endocardium (data not shown). At the mid-ventricle, the onset of sarcomere re-lengthening was sequential in the 3 transmural layers. Namely, re-lengthening first occurred in the epicardium, followed by the mid-myocardium and then the endocardium (Figure 8, arrows indicate the beginning of re-lengthening).

**Figure 9:**
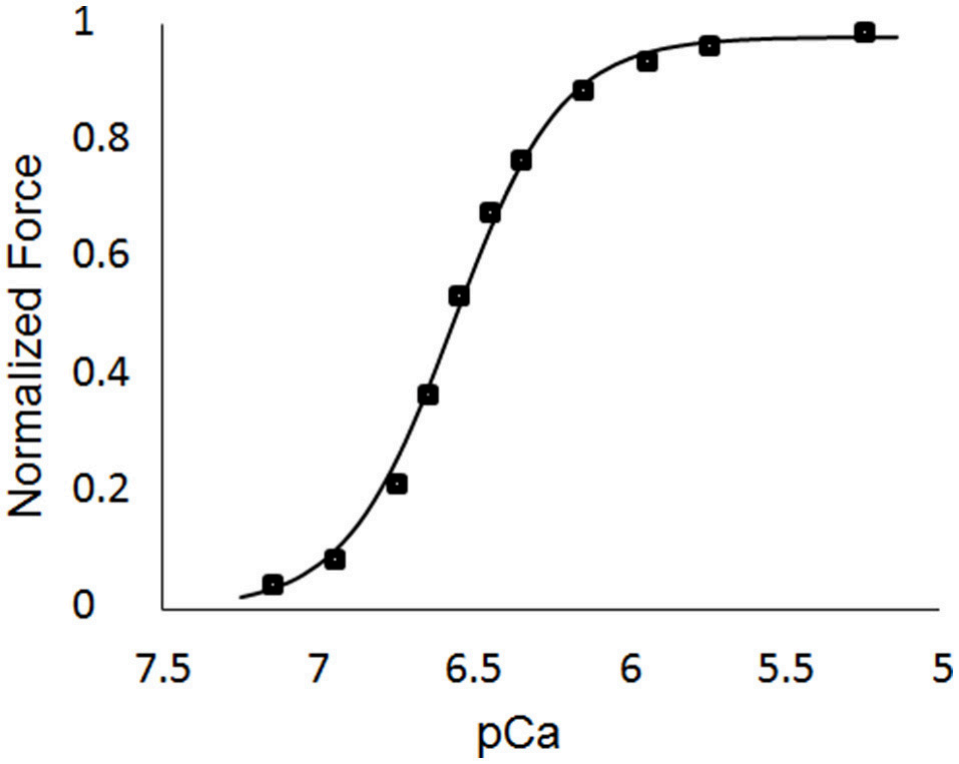
Force-pCa curve for Case 1. The square markers represent the values from the single element simulations, while the curve fit is the Hill equation with a value of *pCa*_50_ = 6.53 and a Hill coefficient = 2.51.

The single element FE model results, which utilized the animal-specific parameters determined from the optimization of the five cases, are shown in Table 3. The maximum tension generation (*T*_*max*_) was found to be 135.3 ± 2.3 kPa. The calcium sensitivity, indicated by the level of calcium at which 50% of the maximum tension is developed (*pCa*_50_), was found to be 6.46 ± 0.024 and the Hill coefficient was 2.41 ± 0.057. The force-pCa curve for Case 1 is shown in Figure 9. The maximum tension redevelopment (*k*_*tr*−*max*_) at saturated calcium was 96.1 ± 1.91 s^−1^ and the minimum tension redevelopment (*k*_*tr*−*min*_) at low calcium was 16.1 ± 1.80 s^−1^.

**Table 3:** Results of the single element simulations of maximum tension generation, calcium sensitivity, and tension redevelopment.

	**Case 1**	**Case 2**	**Case 3**	**Case 4**	**Case 5**
*T*_max_ (*kPa*)	139.5	135.1	126.5	138.5	137
*pCa*_50_	6.53	6.42	6.39	6.48	6.47
*Hill coefficient*	2.51	2.25	2.29	2.53	2.45
*k*_*tr*−*max*_ (s^−1^)	89.5	98.4	101.0	96.3	95.4
*k*_*tr*−*min*_ (s^−1^)	10.3	17.6	21.3	16.5	14.8

## Discussion

In the present study, a novel FE model of active contraction was developed by incorporating a 2-state myocyte contraction model (i.e., MyoSim) into animal-specific ventricle models. This new model includes both length- and velocity-dependence of force generation, and, therefore, has the potential to provide a more accurate representation of cardiac function at the global level, since it is embedded into the 3D FE framework.

Among the several improvements of the new FE contraction model is the inclusion of cooperative effects in dynamic coupling of binding sites and myosin heads. In real muscles, the status of the binding sites influences the status of the myosin heads, and vice versa. This dynamic coupling is further influenced by the status of near neighbor molecules, which is referred to as cooperative effects and is determined by the values of *k*_*plus*_ and *k*_*minus*_. Incorporation of this molecular effect allows the proposed FE model to simulate cardiac muscles with altered Ca^2+^ sensitivity, for example, due to a disease state. This could be assessed by optimizing related parameters (i.e., *a*_*on*_, *a*_*off*_, *k*_*plus*_ and *k*_*minus*_) using experimental data from a diseased animal model, which is beyond the scope of the current study. Moreover, a cross-bridge distribution approach is employed in the proposed FE model to solve the kinetics of binding sites and myosin heads. This approach provides greater control on the strain-dependence of myosin kinetics, enabling the proposed model to mimic the contractile properties of muscles under a wider range of conditions. In addition, the proposed model incorporates the effects of interfilamentary movement. According to Huxley (1957), the length of the cross-bridges changes when the filaments move. The interfilamentary movement, especially at a higher speed relative to the rate of cross-bridge cycling, can perturb the normal cross-bridge distributions, and thus impact the production of active force. This incorporation accounts, at least in part, for the velocity-dependency in the FE model of muscle contraction. Since all of the parameters can be adjusted to fit cellular-level experimental data, this FE contraction model is directly linked to cellular mechanical properties. This is critical, especially when the model is used to relate changes in the mechanical properties of cardiac muscle at the cellular level to variations in the global function of the LV.

In order to validate this new FE contraction model, parameters were optimized to match the experimental data from normal healthy rats. Overall, the proposed model demonstrated strong agreement with the experimental results in terms of LV global function (i.e., PV loop) and regional deformation (i.e., systolic strains and ventricular torsion), although there existed some deviations from experimental measures of strain, mainly in the septal region. Particularly, the notable underestimation of systolic E_ll_ may be due to the assumption of constant fiber angle distribution along the longitudinal direction and could be improved by using more realistic distributions of fiber angles (from DT-MRI for example). The proposed FE model was able to capture the heterogeneity of ventricular relaxation, which has been observed in experiments of intact ventricles *in vivo* (Sengupta et al., 2006; Ashikaga et al., 2007). This is important particularly because the observed delay of sarcomere re-lengthening in the epicardial apex has been associated with pressure drop during early diastole (Sengupta et al., 2006), and therefore may impact diastolic filling. As such, the proposed FE contraction model was able to match experimental PV measurements of not only the systolic phase, but also the diastolic filling phase. This capability could be beneficial in facilitating research related to diastolic dysfunction (Sugiura et al., 2012). Moreover, pilot studies using the time-varying elastance model, a commonly used FE model which does not include a force-velocity relation, failed to optimize the material properties using the same optimization procedure to match the experimental pressure measurements. In addition, these models exhibited synchronized relaxation throughout the entire ventricle. These observations lend further support for the improvement of this new FE model implementation in providing a more accurate representation of LV global function.

It should be noted that some of the optimized values for the active parameters varied from animal to animal, particularly for those used to calculate attachment/detachment rate constants. However, the sensitivity studies previously conducted by Campbell showed that the contraction model (MyoSim) was less sensitive to changes in the strain-dependence of myosin kinetics, as compared to changes in calcium sensitivity which is mediated predominantly by *a*_*on*_ (Campbell, 2014). In line with this, the present study showed similar calcium sensitivity in healthy rats with a narrow range of *a*_*on*_ values. Moreover, although there existed some differences between the optimized values in the present study and those used for unloaded twitch contraction, the maximum proportions of total available binding sites (*N*: 0.15~0.25) and bound myosin heads (*N*_*bound*_: 0.13~0.23) were comparable to the results reported for unloaded twitch contraction (Campbell, 2014). In terms of the single element FE models, which were used to assess *in vivo* cellular-level function, the values for *pCa*_*50*_ were found to be greater than those typically measured with *in vitro* permeabilized cell preparations. However, it has been shown in several studies that intact cells, which are evaluated at *in vivo* temperatures, exhibit greater calcium sensitivity (de Tombe and Stienen, 2007; Chung et al., 2016). Thus, the values found in the present study provide a reasonable representation of *in vivo* values. Additionally, the maximum tension redevelopment (*k*_*tr*−*max*_) at saturated calcium was found to be greater than those measured with *in vitro* experiments. But, it has been shown (de Tombe and Stienen, 2007) that as the temperature of these experiments is increased, the value of *k*_*tr*−*max*_ also increases. Thus, it can be inferred from de Tombe and Stienen (2007) that the values found in the current study are a reasonable estimate of *in vivo* function. The value of the Hill coefficient was found to be lower than those measured from *in vitro* studies (Dobesh et al., 2002; de Tombe and Stienen, 2007), but was still in a range that indicates cooperativitly is in effect. Finally, the maximum tension generated in the current study is in agreement with values found in previous studies (Land et al., 2012).

One limitation of the proposed FE contraction model is the use of a simpler 2-state cross-bridge myosin scheme rather than a more complex 6-state model (Campbell, 2014). However, the 2-state scheme was chosen for a balance between computational efficiency and accuracy. Enhancements to the 2-state model will be pursued in future work. Moreover, the proposed model did not include the effects of cellular shortening on Ca^2+^ transient. But, this effect is relatively small, and the proposed model includes the shortening effects on the total number of binding sites activated by Ca^2+^ as a compensating method. When comparing the FE model strain to the experimental strain, only *n* = 5 samples were used. Finally, the FE models did not include the right ventricle (RV). This likely caused the deformation in the septal region of the FE models to deviate from the experimental measurements from CMR. To overcome this limitation, the RV will be included in future models.

In conclusion, the proposed FE contraction model successfully predicted both the global function and regional deformation of the LV. The capability of the proposed model to capture an important feature of ventricular relaxation makes it a powerful tool for the future investigation of diastolic dysfunction. Moreover, the incorporation of cellular-level mechanisms may enable the proposed model to assess how pharmaceutical treatments that target cellular function influence global ventricular function.

## Author contributions

XZ wrote the finite element code, performed the computational analysis, collected the experimental data, and wrote most of the manuscript. Z-QL analyzed the experimental data in terms of 3D surface generation and regional strain. KC developed the cellular-level code and helped implement it into the finite element framework. JW helped implement the finite element code, analyze both the experimental/computational results, write the manuscript, and developed the conceptual design of the work.

### Conflict of interest statement

The authors declare that the research was conducted in the absence of any commercial or financial relationships that could be construed as a potential conflict of interest.

## References

[B1] Ashikaga H. Coppola B. A. Hopenfeld B. Leifer E. S. Mcveigh E. R. Omens J. H. (2007). Transmural dispersion of myofiber mechanics: implications for electrical heterogeneity *in vivo*. J. Am. Coll. Cardiol. 49, 909–916. 10.1016/j.jacc.2006.07.074 17320750 PMC2572715

[B2] Campbell K. S. (2009). Interactions between connected half-sarcomeres produce emergent mechanical behavior in a mathematical model of muscle. PLoS Comput. Biol. 5:e1000560. 10.1371/journal.pcbi.1000560 19911050 PMC2770126

[B3] Campbell K. S. (2014). Dynamic coupling of regulated binding sites and cycling myosin heads in striated muscle. J. Gen. Physiol. 143, 387–399. 10.1085/jgp.201311078 24516189 PMC3933939

[B4] Campbell S. G. Flaim S. N. Leem C. H. Mcculloch A. D. (2008). Mechanisms of transmurally varying myocyte electromechanics in an integrated computational model. Philos. Trans. A Math. Phys. Eng. Sci. 366, 3361–3380. 10.1098/rsta.2008.0088 18593662 PMC2556206

[B5] Chung J. H. Biesiadecki B. J. Ziolo M. T. Davis J. P. Janssen P. M. (2016). Myofilament calcium sensitivity: role in regulation of *in vivo* cardiac contraction and relaxation. Front. Physiol. 7:562. 10.3389/fphys.2016.00562 28018228 PMC5159616

[B6] Daniels M. Noble M. I. Ter Keurs H. E. Wohlfart B. (1984). Velocity of sarcomere shortening in rat cardiac muscle: relationship to force, sarcomere length, calcium and time. J. Physiol. 355, 367–381. 10.1113/jphysiol.1984.sp015424 6491996 PMC1193496

[B7] de Tombe P. P. Stienen G. J. (2007). Impact of temperature on cross-bridge cycling kinetics in rat myocardium. J. Physiol. 584, 591–600. 10.1113/jphysiol.2007.138693 17717017 PMC2277159

[B8] Dobesh D. P. Konhilas J. P. De Tombe P. P. (2002). Cooperative activation in cardiac muscle: impact of sarcomere length. Am. J. Physiol. Heart Circ. Physiol. 282, H1055–H1062. 10.1152/ajpheart.00667.2001 11834504

[B9] Gordon A. M. Homsher E. Regnier M. (2000). Regulation of contraction in striated muscle. Physiol. Rev. 80, 853–924. 10.1152/physrev.2000.80.2.853 10747208

[B10] Guccione J. M. McCulloch A. D. (1993). Mechanics of active contraction in cardiac muscle: Part I–Constitutive relations for fiber stress that describe deactivation. J. Biomech. Eng. 115, 72–81. 10.1115/1.2895473 8445901

[B11] Guccione J. M. Moonly S. M. Moustakidis P. Costa K. D. Moulton M. J. Ratcliffe M. B.. (2001). Mechanism underlying mechanical dysfunction in the border zone of left ventricular aneurysm: a finite element model study. Ann. Thorac. Surg. 71, 654–662. 10.1016/S0003-4975(00)02338-9 11235723

[B12] Guccione J. M. Waldman L. K. McCulloch A. D. (1993). Mechanics of active contraction in cardiac muscle: Part II–Cylindrical models of the systolic left ventricle. J. Biomech. Eng. 115, 82–90. 10.1115/1.2895474 8445902

[B13] Haggerty C. M. Kramer S. P. Binkley C. M. Powell D. K. Mattingly A. C. Charnigo R.. (2013). Reproducibility of cine displacement encoding with stimulated echoes (DENSE) cardiovascular magnetic resonance for measuring left ventricular strains, torsion, and synchrony in mice. J. Cardiovasc. Magn. Reson. 15:71. 10.1186/1532-429X-15-71 23981339 PMC3765995

[B14] Hunter P. J. McCulloch A. D. Ter Keurs H. E. (1998). Modelling the mechanical properties of cardiac muscle. Prog. Biophys. Mol. Biol. 69, 289–331. 10.1016/S0079-6107(98)00013-3 9785944

[B15] Huxley A. F. (1957). Muscle structure and theories of contraction. Prog. Biophys. Biophys. Chem. 7, 255–318. 13485191

[B16] Huxley H. E. Stewart A. Sosa H. Irving T. (1994). X-ray diffraction measurements of the extensibility of actin and myosin filaments in contracting muscle. Biophys. J. 67, 2411–2421. 10.1016/S0006-3495(94)80728-3 7696481 PMC1225626

[B17] Kerckhoffs R. C. Neal M. L. Gu Q. Bassingthwaighte J. B. Omens J. H. Mcculloch A. D. (2007). Coupling of a 3D finite element model of cardiac ventricular mechanics to lumped systems models of the systemic and pulmonic circulation. Ann. Biomed. Eng. 35, 1–18. 10.1007/s10439-006-9212-7 17111210 PMC2872168

[B18] Kobayashi T. Solaro R. J. (2005). Calcium, thin filaments, and the integrative biology of cardiac contractility. Annu. Rev. Physiol. 67, 39–67. 10.1146/annurev.physiol.67.040403.114025 15709952

[B19] Land S. Niederer S. A. Aronsen J. M. Espe E. K. Zhang L. Louch W. E.. (2012). An analysis of deformation-dependent electromechanical coupling in the mouse heart. J. Physiol. 590, 4553–4569. 10.1113/jphysiol.2012.231928 22615436 PMC3477757

[B20] Laurita K. R. Singal A. (2001). Mapping action potentials and calcium transients simultaneously from the intact heart. Am. J. Physiol. Heart Circ. Physiol. 280, H2053–H2060. 10.1152/ajpheart.2001.280.5.H2053 11299206

[B21] Pacher P. Nagayama T. Mukhopadhyay P. Batkai S. Kass D. A. (2008). Measurement of cardiac function using pressure-volume conductance catheter technique in mice and rats. Nat. Protoc. 3, 1422–1434. 10.1038/nprot.2008.138 18772869 PMC2597499

[B22] Razumova M. V. Bukatina A. E. Campbell K. B. (1999). Stiffness-distortion sarcomere model for muscle simulation. J. Appl. Physiol. 87, 1861–1876. 10.1152/jappl.1999.87.5.1861 10562631

[B23] Rice J. J. Wang F. Bers D. M. De Tombe P. P. (2008). Approximate model of cooperative activation and crossbridge cycling in cardiac muscle using ordinary differential equations. Biophys. J. 95, 2368–2390. 10.1529/biophysj.107.119487 18234826 PMC2517033

[B24] Rüssel I. K. Tecelao S. R. Kuijer J. P. Heethaar R. M. Marcus J. T. (2009). Comparison of 2D and 3D calculation of left ventricular torsion as circumferential-longitudinal shear angle using cardiovascular magnetic resonance tagging. J. Cardiovasc. Magn. Reson. 11:8. 10.1186/1532-429X-11-8 19379480 PMC2689859

[B25] Schneider N. S. Shimayoshi T. Amano A. Matsuda T. (2006). Mechanism of the Frank-Starling law–a simulation study with a novel cardiac muscle contraction model that includes titin and troponin I. J. Mol. Cell. Cardiol. 41, 522–536. 10.1016/j.yjmcc.2006.06.003 16860336

[B26] Sengupta P. P. Khandheria B. K. Korinek J. Wang J. Jahangir A. Seward J. B.. (2006). Apex-to-base dispersion in regional timing of left ventricular shortening and lengthening. J. Am. Coll. Cardiol. 47, 163–172. 10.1016/j.jacc.2005.08.073 16386681

[B27] Spottiswoode B. S. Zhong X. Hess A. T. Kramer C. M. Meintjes E. M. Mayosi B. M.. (2007). Tracking myocardial motion from cine DENSE images using spatiotemporal phase unwrapping and temporal fitting. IEEE Trans. Med. Imaging 26, 15–30. 10.1109/TMI.2006.884215 17243581

[B28] Sugiura S. Washio T. Hatano A. Okada J. Watanabe H. Hisada T. (2012). Multi-scale simulations of cardiac electrophysiology and mechanics using the University of Tokyo heart simulator. Prog. Biophys. Mol. Biol. 110, 380–389. 10.1016/j.pbiomolbio.2012.07.001 22828714

[B29] Tajima Y. Makino K. Hanyuu T. Wakabayashi K. Amemiya Y. (1994). X-ray evidence for the elongation of thin and thick filaments during isometric contraction of a molluscan smooth muscle. J. Muscle Res. Cell Motil. 15, 659–671. 10.1007/BF00121073 7706422

[B30] Keurs H. E. Bucx J. J. De Tombe P. P. Backx P. Iwazumi T. (1988). The effects of sarcomere length and Ca++ on force and velocity of shortening in cardiac muscle. Adv. Exp. Med. Biol. 226, 581–593. 3407533

[B31] ter Keurs H. E. Rijnsburger W. H. Van Heuningen R. Nagelsmit M. J. (1980). Tension development and sarcomere length in rat cardiac trabeculae. Evidence of length-dependent activation. Circ. Res. 46, 703–714. 10.1161/01.RES.46.5.703 7363419

[B32] Trayanova N. A. Rice J. J. (2011). Cardiac electromechanical models: from cell to organ. Front. Physiol. 2:43. 10.3389/fphys.2011.00043 21886622 PMC3154390

[B33] Trumble D. R. McGregor W. E. Kerckhoffs R. C. Waldman L. K. (2011). Cardiac assist with a twist: apical torsion as a means to improve failing heart function. J. Biomech. Eng. 133:101003. 10.1115/1.4005169 22070328

[B34] Wang H. Zhang X. Dorsey S. M. McGarvey J. R. Campbell K. S. Burdick J. A.. (2016). Computational investigation of transmural differences in left ventricular contractility. J. Biomech. Eng. 138, 114501. 10.1115/1.4034558 27591094 PMC5125313

[B35] Wenk J. F. Klepach D. Lee L. C. Zhang Z. Ge L. Tseng E. E.. (2012). First evidence of depressed contractility in the border zone of a human myocardial infarction. Ann. Thorac. Surg. 93, 1188–1193. 10.1016/j.athoracsur.2011.12.066 22326127 PMC3314154

[B36] Wenk J. F. Sun K. Zhang Z. Soleimani M. Ge L. Saloner D.. (2011). Regional left ventricular myocardial contractility and stress in a finite element model of posterobasal myocardial infarction. J. Biomech. Eng. 133:044501. 10.1115/1.4003438 21428685 PMC3097530

[B37] Zhang X. Haynes P. Campbell K. S. Wenk J. F. (2015). Numerical evaluation of myofiber orientation and transmural contractile strength on left ventricular function. J. Biomech. Eng. 137:044502. 10.1115/1.4028990 25367232 PMC5101031

[B38] Zhang X. Liu Z. Q. Singh D. Wehner G. J. Powell D. K. Campbell K. S.. (2017). Regional quantification of myocardial mechanics in rat using 3D cine DENSE cardiovascular magnetic resonance. NMR Biomed. 30:e3733. 10.1002/nbm.3733 28481037 PMC10539034

[B39] Zhong X. Gibberman L. B. Spottiswoode B. S. Gilliam A. D. Meyer C. H. French B. A.. (2011). Comprehensive cardiovascular magnetic resonance of myocardial mechanics in mice using three-dimensional cine DENSE. J. Cardiovasc. Magn. Reson. 13:83. 10.1186/1532-429X-13-83 22208954 PMC3278394

